# Fabrication of Interconnected Plasmonic Spherical Silver Nanoparticles with Enhanced Localized Surface Plasmon Resonance (LSPR) Peaks Using Quince Leaf Extract Solution

**DOI:** 10.3390/nano9111557

**Published:** 2019-11-02

**Authors:** Shujahadeen B. Aziz, Govar Hussein, M. A. Brza, Sewara J. Mohammed, R. T. Abdulwahid, Salah Raza Saeed, Abdollah Hassanzadeh

**Affiliations:** 1 Prof. Hameeds Advanced Polymeric Materials Research Lab., Department of Physics, College of Science, University of Sulaimani, Qlyasan Street, Sulaimani 46001, Iraq; mohamad.brza@gmail.com (M.A.B.); rebar.abdulwahid@univsul.edu.iq (R.T.A.); 2 Komar Research Center (KRC), Komar University of Science and Technology, Sulaimani 46001, Iraq; 3 Department of Physics, University of Kurdistan, Sanandaj, Kurdistan, Iran; govar.hussein@gmail.com (G.H.); a.hassanzadeh@uok.ac.ir (A.H.); 4 Department of Manufacturing and Materials Engineering, Faculty of Engineering, International Islamic University of Malaysia, Kuala Lumpur, Gombak 53100, Malaysia; 5 Department of Chemistry, College of Science, University of Sulaimani, Qlyasan Street, Sulaimani 46001, Iraq; sewara.mohammed@univsul.edu.iq; 6 Charmo Research Center, Charmo University, Peshawa Street, Chamchamal, Sulaimani 46001, Iraq; salah1966@gmail.com

**Keywords:** quince leave, silver nanoparticles, UV–Vis absorption, FTIR study, XRD analysis, FESEM study

## Abstract

Interconnected spherical metallic silver nanoparticles (Ag NPs) were synthesized in the current study using a green chemistry method. The reduction of silver ions to Ag NPs was carried out with low-cost and eco-friendly quince leaves. For the first time, it was confirmed that the extract solution of quince leaves could be used to perform green production of Ag NPs. Fourier transform infrared spectroscopy (FTIR) was conducted to identify the potential biomolecules that were involved in the Ag NPs. The results depicted that the biosynthesis of Ag NPs through the extract solution of quince leaf was a low-cost, clean, and safe method, which did not make use of any contaminated element and hence, had no undesirable effects. The majority of the peaks in the FTIR spectrum of quince leaf extracts also emerged in the FTIR spectrum of Ag NPs but they were found to be of less severe intensity. The silver ion reduction was elaborated in detail on the basis of the FTIR outcomes. In addition, through X-ray diffraction (XRD) analysis, the Ag NPs were also confirmed to be crystalline in type, owing to the appearance of distinct peaks related to the Ag NPs. The creation of Ag NPs was furthermore confirmed by using absorption spectrum, in which a localized surface plasmon resonance (LSPR) peak at 480 nm was observed. The LSPR peak achieved in the present work was found to be of great interest compared to those reported in literature. Field emission scanning electron microscopy (FESEM) images were used to provide the morphology and grain size of Ag NPs. It was shown from the FESEM images that the Ag NPs had interconnected spherical morphology.

## 1. Introduction

The branch of technology that focuses on atomic, molecular, as well as supramolecular molecules is nanotechnology, which seeks to generate nanostructures having improved functionalities. Nanoparticles are particulate matter that have sizes approximately from 1 to 100 nm. The nanoscale size means that they have a considerable ratio of large surface area to volume [1]. It is possible for noble metal nanoparticles to exhibit excellent and tunable optical characteristics because of the surface plasmon resonance (SPR) [2]. Excellent physiochemical, biochemical, and optoelectronic properties are demonstrated by the noble-metal nanoparticles. Industrial and pharmaceutical applications extensively make use of these nanoparticles [3]. Even though several metals are present in the environment, there are very few that are mainly synthesized in nanostructured forms, for example as gold, silver, platinum, and palladium [3,4,5]. In the last few decades, scientists have given a lot of attention to the studies pertaining to advanced nanomaterials of noble metals such as silver, because these metals have physiochemical properties like distribution, size, and morphology. These metals have been assessed for their catalytic activity and their electronic, optical, antibacterial, and magnetic characteristics [6]. Distinct optical properties are exhibited by noble metal nanoparticles, for example resonant absorption and scattering of light as a result of the combined free electron excitations in the conduction band, which is lacking in bulk correspondents [7]. A strong absorption band is created because of the SPR phenomenon, which typically centers on the visible range. In addition, this phenomenon also creates the largely effective third-order nonlinear optical susceptibility frequently measured, and which is attributable to the powerful improvement in the local electric field that is closest to the nanoclusters. As a result, these materials may possibly be used in optoelectronic devices like ultrafast optical switches [8]. At present, SPRs within noble metal nanoparticles are being used presently for several purposes, for example molecular sensing, molecular tagging, focusing light, near-field scanning optical microscopy (NSOM) as well as subwavelength photonics. The demand of surface plasmon excitations for such uses because of the wide enhancement in the electromagnetic field close to the surface of the metals as well as the fact that the wavelength at resonance is dependent on the size, shape, as well as local dielectric of the nanoparticles’ surroundings [9]. As nanotechnology is used in various applications, it is important to develop novel and successful methods to develop the nanoparticles.

Different physical and chemical techniques are used for the creation of nanoparticles, for example UV irradiation, microwave irradiation chemical reduction [10], pyrolysis, laser ablation, chemical vapour deposition, physical vapour deposition, sol–gel [11], and so on. Organic solvents for example ethanol, N,N/-dimethyl formamide, and ethylene glycol [12,13,14,15] are employed to reduce silver ions to Ag nanoparticles (NPs) in addition to other reducing agents for instance sodium borohydride, hydrazine hydrate, glucose, and sodium formaldehyde sulfoxylate [16,17,18,19].

Viorica et al. [20] fabricated nanocomposite thin films based on zinc oxide (ZnO) nanoparticle and Ag NPs/ZnO doped in chitosan (CS) polymer as well as integrated in poly(methyl methacrylate) (PMMA) polymer through a modified sol–gel technique. The nanocomposite thin films of Ag:ZnO/CS/PMMA demonstrate low surface roughness, large optical transmittance of around 90%, high dielectric constant in the range of 9.2 to 9.5 at 20 kHz, as well as optical band gap values in the range of 3.543 to 3.737 eV. The nanocomposite film has been investigated for possible applications in various fields such as optics, photonics, as well as electronics.

Pimentel et al. [21] synthesized zinc oxide nanorods (ZnO NRs) arrays through a hydrothermal technique supported by radiation of microwave on cardboard materials substrates coated with a ZnO seed thin film. The ZnO NRs were decorated with Ag NPs. Synthesis times between 5 and 30 min as well as temperatures between 70 and 130 °C were tested. It is observed that the ZnO NRs diameter and length increase with increasing synthesis time and temperature while their optical band gap energy decreases, signifying an enlargement in crystallite size. Furthermore, Ag NPs were deposited on the ZnO NRs, therefore it was possible to test the provided substrates as platforms for the application of surface-enhanced Raman scattering.

Several problems existed with these above methods, for example problems in purification, toxicity, low material conversions, extensive energy requirements, expensive, and dangerous chemicals, which is why researchers have shifted to the green synthesis technique, which is an environmentally-friendly method [10,11,22,23,24]. There are two forms of the green synthesis technique: biological microorganisms and plant extracts. A more simple and robust method of creating the nanoparticles is plant extract [25]. *Camellia sinensis*, *Ocimum*, *Arbutus Unedo*, and *Rosa rugosa* are the plants used most commonly [26,27,28]. Nanoscience acknowledges Ag NPs to a large extent, which has several uses in our life because of the impeccable physical and chemical qualities, for example excellent thermal and electrical conductivity, catalytic impact [29,30], as well as because of the medical and biological properties, for example, anti-fungal, anti-bacterial, anti-inflammatory, as well as anti-viral characteristics [10,31]. In an analysis carried out recently by Roy et al. [32], it was claimed that out of the different nanoparticles, a greater degree of attention is now being given to Ag NPs because they have distinct antimicrobial properties. Nonetheless, because of the issues regarding these materials’ production, like the use of precursor chemicals, poisonous solvents, and the creation of poisonous byproducts, a unique substitute method has been put forward, referred to as green synthesis. This method is eco-friendly and includes the employ of plants, biological or microbial agents, such as reducing agents and capping agents. An innovative and possible alternative for the chemically generated nanoparticles is the production of Ag NPs through green chemistry [32]. Ag NPs are developed in the current study using green techniques. An enhanced platform is offered for the creation of nanoparticles by plants since they do not include any toxic chemicals and also include natural capping agents [6]. Several studies performed in the past few decades have shown the chemical structure and biological impact of quince. Bioactive phytochemicals are present in the leaves of quince, which contain phenolic compounds [33] that have several conjugated double bonds. Although the exact process through which nanoparticles biosynthesis is carried out by plant extracts is not clear, biomolecules in plant extracts like phenol, protein, and flavonoids [3,34,35,36] have been found to play an important part in metal ions’ reduction and the capping of biosynthesized nanoparticles [3,34]. In this study, quince leaf was selected out of the different plants available.

## 2. Experimental Methodology

### 2.1. Synthesis of Silver Nanoparticles (Ag NPs)

In this work, Ag NPs were fabricated using the green method. In this regard, quince leaves were employed to attain natural colorants that were included through phenolic compounds. The quince is the sole *Cydonia* group member in the Rosaceae family and is a deciduous tree that develops a pome fruit that is identical to a pear, and has an intense yellow colour when it has grown [33]. Unfermented quince leaves were used to achieve the objectives of the current study. The provided green quince leaves were washed using distilled water. They were then dried at ambient temperature, after which they were kept safe from being exposed to sunlight for seven days. The quince leaves were then extracted of their natural colourant. This was carried out by adding 4g of quince leaf to 400 mL distilled water at 80 °C, while not allowing them to be under sun exposure. The steady temperature was maintained for the solution. Filtration was carried out to eliminate the residues, and for this Whatman filter paper with a 20 µm pore size was employed. Following this, 0.1 g of dissolved silver nitrate (AgNO_3_) in distilled water (30 mL) was added to the extracted solution of quince leaves for approximately six minutes. The creation of Ag NPs was evident when the solution colour became dark brown. There silver ions reduced to Ag NPs within the medium of conjugated double bonds of phenolic compounds in the leaves of quince. The reaction occurred between the Ag^+1^ cations and quercetin as reducing agent in the quince leaves extract solution at a temperature around 80 °C to synthesize Ag NPs as demonstrated in Section 3.1 in detail. Once the solution temperature reached room temperature, a centrifuge machine was used for Ag NPs separation and precipitation. The precipitated Ag NPs were washed around five times using distilled water repetitively. The end process was Ag NPs dispersed in 50 mL of distilled water. The suspended Ag NPs pH was 6.

### 2.2. X-ray Diffraction

An X-ray diffractometer (Empyrean XRD-Panalytical, Netherland) was used to record the X-ray diffraction (XRD) at ambient temperature, with a working voltage of 40 kV and current of 45 mA. A monochromatic beam, X-ray radiation, with a wavelength λ = 1.5406 Å was employed to examine the deposited Ag NPs on a glass slide and the glancing angles were between 5° ≤ 2θ ≤ 90°, with a 0.05° step size.

### 2.3. Fourier Transform Infrared (FTIR) Spectroscopy 

The silver nanoparticles and quince leaves were examined using FTIR spectrophotometer (Thermo Fischer Scientific, Waltham, MA, USA), in the wavenumber range between 4000 and 400 cm^−1^ and 2 cm^−1^ resolution. The dry powders of quince leaves and Ag NPs were used for FTIR study.

### 2.4. Ultraviolet–Visible (UV-Vis) Measurement

A Jasco V-570 UV–Vis-NIR spectrophotometer (Jasco SLM-468, Tokyo, Japan) operating in the absorbance mode was used to study the Ag NPs UV–Vis absorption spectra. The diluted solution of Ag NPs was inserted into the cuvette for UV–Vis measurement.

## 3. Results and Discussion

### 3.1. FTIR Analysis and Mechanism of Ag^+1^Ion Reduction

**Figure 1:**
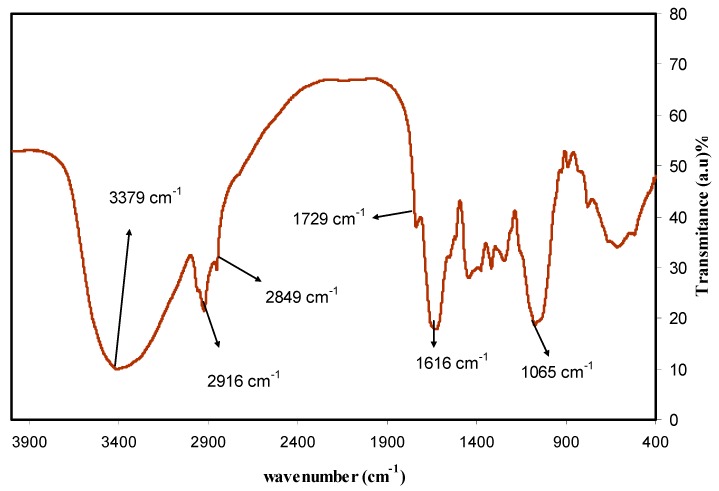
Spectra of pure quince leaves.

The biomolecules of quince leaf were distinguished and recognized using FTIR spectroscopy. Several researchers have used FTIR to analyse different materials. Information is provided by FTIR spectroscopy regarding intermolecular interaction by examining FTIR spectra based on stretching or bending vibration of specific bonds [37]. The FTIR spectrum of the quince leaf is presented in Figure 1. At 3379 cm^−1^, an intense broad band was observed, which was due to O–H stretching modes of polyphenols [10,22]. The extensive absorption band at 3310.7 cm^−1^ was also ascribed by Saif et al. [38] to the hydroxyl (OH) group within phenolic compounds as well as alcohols. Another powerful band at 1616 cm^−1^ may also have been due to the C=C stretch vibration in the aromatic ring, as well as the C=O stretch vibration in polyphenols [22,39]. It has been found that the C–H and O–H stretches in alkanes and carboxylic acid surface at 2916 and 2849 cm^−1^, respectively [22]. A band at 1065 cm^−1^ has also emerged because of the C–O bond stretching in amino acid [22,39]. It was also shown in the latest studies that there were identical FTIR bands in different kinds of tea, such as black, green, and oolong tea [36,40]. Previous studies demonstrated that the FTIR bands emerged at 3388 cm^−1^, 1636 cm^−1^, as well as 1039 cm^−1^, are ascribed to O–H/N–H, C=C, and C–O–C stretching vibrations, correspondingly [22,36,39,41,42]. Hence, it can be noted in the infrared spectra that the major functional groups in the quince leaf extract solution were carboxylic acid, polyphenols, and amino acid. It has been deduced that colloidal suspensions were a result of the interaction of polyphenols with the cations of silver metals [43]. Hence, the idea of the recent study was basically to exhibit that silver colloidal nanoparticles related to metallic silver particles can be productively identified from the FTIR analysis. Understanding the way interconnected metallic silver particles were developed by green techniques was fairly easy from the point of view of chemistry and physics, because there were widespread amount of polyphenols and conjugated double bonds in the extract solution of quince leaf, as demonstrated by FTIR analysis, and the way those components interact with silver salt as a means of reducing and capping of silver ions (Ag^+1^). In addition, the formation of colloidal suspension within the beaker verified the formation of Ag NPs between silver cations and polyphenols. The optical absorption behaviour of silver metal colloidal suspension will be discussed in Section 3.3. Normally, strong absorption is demonstrated by silver metal nanoparticles in visible light ranges. From the point of view of physics, distinguishing bulk size metallic particles from nano-sized particles is based on light absorption. Hence, an optical absorption study was carried out in Section 3.3 to understand the nature of colloidal suspension of silver metal nanoparticles. 

**Figure 2:**
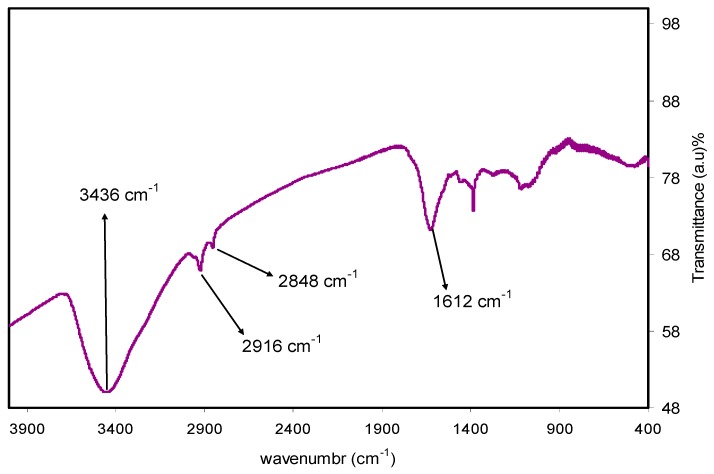
FTIR spectra for silver metal nanoparticles.

**Figure 3:**
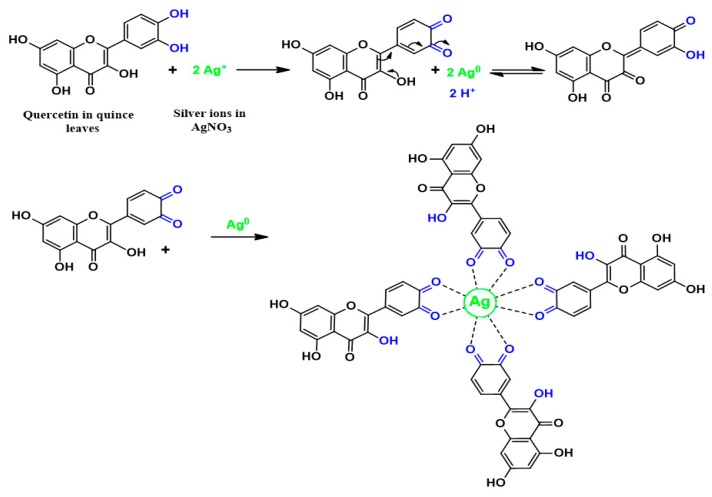
Mechanism for the silver ions reduction to metallic silver nanoparticles via quercetin as reducing agent in quince leaves extract solution.

Based on Figure 1 and Figure 2 the mechanism of silver ion reduction can be grasped easily. The comparison of FTIR bands appeared in Figure 2 to those presented in Figure 1 was supportive to understand the mechanism of silver ion reduction and to assume some scheme about silver ion reduction in a mediated polyphenol solution. It was determined in the past few studies that quince leaves consist of quercetin and kaempferol derivatives that were determined in peels as well as leaves: quercetin-3-O-galactoside, quercetin-3-O-rutinoside, kaempferol-3-O-glucoside, as well as kaempferol-3-O-rutinoside [44]. It was elaborated by Xu et al. [45] in a recent study that polyphenols can function as multi-talented foundation for the different functional materials creation, for example, antibacterial films, antioxidant films, capsules, membranes, micro particles, nanoparticles, hydrogels, electronic materials, energy storage materials, as well as cell encapsulants, which have interesting properties and structures. It is demonstrated in the latest reviews of metal complexes of Cu and Fe that these ions bind to the two groups –NH_2_ and –OH [46]. A former study showed that polyphenols are gifted to chelate ionic metal (for instance iron and copper), which can bring about the conclusion that antioxidant activity depends on polyphenol chelation of metal ions, because this prevents redox-active transition metals from catalysing the development of free radicals [47]. There are no previous studies that elaborate on the mechanism used to cause flavonoid reduction and stabilization of Ag NPs. There are effectively three stages in which nanoparticle creation takes place: ions reduction, clustering, and subsequent growth of the nanoparticle [48]. The reduction and oxidation reactions of aqueous Ag NO_3_ solutions mediated by quince leave solution at temperature (80 °C) led to the creation of silver-based nanostructures. It was shown in the latest studies that the caffeine spectrum went through changes in the range between 1700 and 400 cm^−1^, as can be seen in Figure 1, in accordance with the stretching vibration and binding vibration of imidazole, carbonyl, pyrimidine, as well as methyl fragments within the caffeine [49,50]. The intensity of the band at 1065 cm^−1^ (shown in Figure 1) almost disappeared in the FTIR spectra of Ag NPs as shown in Figure 2. By comparing Figure 1 and Figure 2, it can be seen that the peaks that occurred in the (1700–400) cm^−1^ region nearly underwent modification in Figure 2 with weak intensities. The FTIR spectrum of Ag NPs in Figure 2 shows that the wavenumber of the OH bending vibration at 3436 cm^-1^ seemed to widen and decrease in intensity. There was a shift in the peak of the C=O stretching vibration of carboxylic acid groups at 1616 cm^−1^ (see Figure 1), and it seems that the intensity of this bands decreases and shifts to 1612 cm^-1^ (shown in Figure 2). It is indicated by the suppression of the bands related to the caffeine and other band vibrations that biomolecules of extract solution of quince leaves captured the silver ions and reduced them to metallic silver particles. These kinds of organic capping may be developed because of the preliminary reduction of the silver ions by means of their complexations with the functional groups of the polyphenols [51]. This is because when coordination takes place between Ag^+1^cations, polyphenols, and caffeine, their vibrations decrease because of the attachment of Ag^+1^cations and hence, there is an increase in their molecular weight. It was determined in more advanced research that quince leaf extracts are essentially made up of polyphenols that are enriched by functional groups like OH [52,53]. Therefore, on the basis of earlier studies [44,45,48,54] and FTIR analysis of the current study, Figure 3 presents the suggested scheme for silver ion reduction using the flavonoids of quercetin of quince leaf extract solution. Using density functional theory (DFT) analysis, it was shown in an earlier study that there are lower O–H bond dissociation energies of –OH groups of the catechol moiety of flavonoids of quercetin of quince leaf extract solution compared to other –OH groups existing in flavonoids [48]. The proposed structure for the Ag-complex development clearly indicated that Ag^+1^was able to form complexationwith quercetin (refer to Figure 3). According to the previous study, there were ample conjugated double bonds, hydroxyl groups, carboxylic groups, polyphenols, as well as polyphenol conjugates in the extract solutions of green leaves [35,36].Hence, Ag^+1^cation reduction is coupled with the oxidation of hydroxyl group of polyphenols [55]. Commonly, the Ag NPs formation mechanism in the existence of flavonoids lies at the group of diorthohydroxyl in their B-ring that enhances the Ag^+1^cation reduction to Ag NPs via the release of two electrons [54].

The reason why there was dispersion and stabilization of Ag NPs as interconnected silver particles was the presence of the chemical bond among the electron rich oxygen that exists in the quercetin macromolecule of the flavonoids and the silver orbital through their sole pair electrons [55]. The complex creation of metal cations with the polyphenols of extract black tea solution was also demonstrated by Goodman et al. [56] by examining the electron paramagnetic resonance (EPR) method. The creation of Ag NPs was depicted in the current study using the FTIR method. The Ag NPs FTIR spectra observed in the current study is quite similar to the FTIR spectra determined by Kaur and Jaryal [57] who synthesized AgNPs through biogenic tea waste. A UV–Vis study on Ag NPs was conducted to obtain additional confirmation, which is shown in the following section. Ag NPs are known to have good absorption and to exhibit localized surface plasmon resonance (LSPR) peaks within the visible region.

### 3.2. XRD Analysis

**Figure 4:**
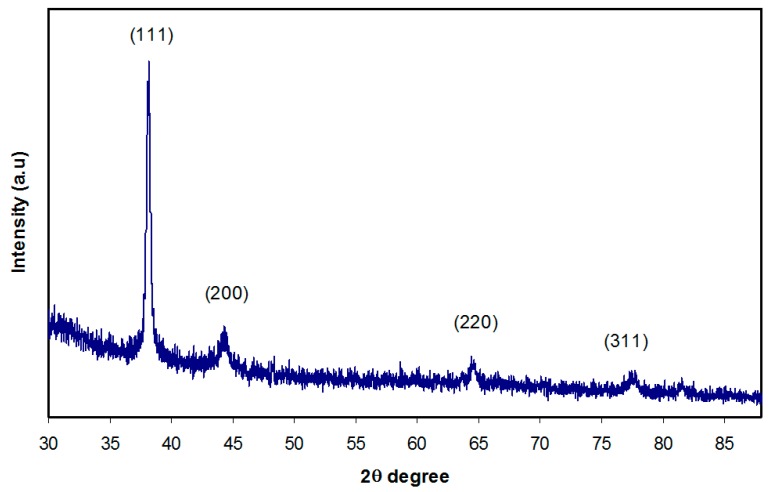
Pattern of interconnected spherical Ag nanoparticles.

XRD was measured for the sample to substantiate the creation of Ag NPs in quince leave extract solution. Using the XRD, the Ag NPs crystalline size and structure were examined. Figure 4 demonstrates the XRD spectrum of Ag NPs. The XRD pattern attained is demonstrated in Figure 4, and it is evident that the quince leave extract solution caused Ag^+1^cation reduction to Ag NPs, as distinct peaks emerge from 2θ =30° to 80°. Different peaks emerged at 2θ values of 38°, 44.67°, 65.08°, and 78.06°, which were relative to the (111), (200), (220), and (311) planes of the face-centred cubic (FCC) of Ag NPs, correspondingly [58]. Therefore, it was evident from the XRD pattern in Figure 4 that the FCC form of metallic Ag NPs was present and there were without peaks of other impurity crystalline phases [58,59,60,61]. The intensity of the peaks relevant to the (111) plane is quite high compared to the rest of the planes. These peaks become wider mainly because of the impact of nano-sized particles [62]. The XRD patterns and peaks of Ag NPs are quite similar to those observed in previous studies that utilize various plant extracts [63,64]. The reason for this is that the functional groups of reduction media serve as vital capping reagents that are effective in stabilizing the synthesized Ag NPs [58]. The slight shift of the peak positions may be related to the possible stresses connected to agglomerated/clusters formed.

### 3.3. UV–Visible Absorption Study

**Figure 5:**
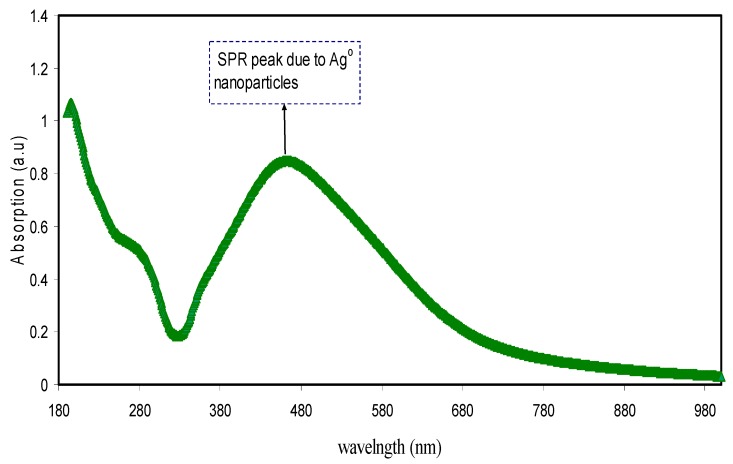
Absorption spectra for colloidal Ag nanoparticles.

The UV–Vis absorption spectra pertaining to the synthesized metallic Ag NPs using green method is demonstrated in Figure 5. The visible region shows a distinct LSPR. The SPR emerged at around 480 nm verified the Ag NPs creation. The optical response of metal particles in nano ranges was confirmed to usually be distinguished through the existence of a powerful absorption and scattering peak that was absent in the bulk metal spectra. This is because of the combining of the conduction electrons within the metal to the incident light electromagnetic field [65]. When visible light at a relevant frequency passes through a noble metal nanoparticle, the conduction electrons that are spatially confined go through a coherent oscillation that is referred to as the LSPR. The geometry of the particle regulates the LSPR spectral position and thickness, as well as the dielectric functions of both metals and nearby media, inter-particle interactions, and incident light polarization [66]. The conduction electrons oscillate as a reaction to the electromagnetic waves, generating an electric field around the surface which has a small penetration depth [67]. The peaks may be caused by the LSPR excitation which takes place because of the nanoscale-size metal particles [68]. It is shown by the experimental results that a very intense colour shown by the metal clusters that was not evident in the materials in bulk or in the individual atoms. This is because of the combined oscillation of the conduction electrons brought about through the interacting electromagnetic field. These resonances are also referred to as surface plasmons [69]. The broad absorption which covered almost the entire range of visible by Ag NPs in the present work is crucial from the viewpoint of plasmonic solar cells.

Dispersion of Ag NPs in Polyvinyl alcohol PVA polymer by the use of silver cation in situ reduction via two various reducing agents, specifically hydrazine hydrate as well as sodium formaldehyde sulfoxylate, has been documented by Khanna et al. [19] UV–Vis spectroscopy was used to comprehend the Ag NPs property provided using the two reducing agents. The Ag NPs SPR peaks synthesized by means of hydrazine hydrate as well as sodium formaldehyde sulfoxylate within the PVA polymer were at around 418 nm, signifying Ag NPs development in the PVA matrix. Nevertheless, the absorption spectrum was sharp for Ag NPs formed using sodium formaldehyde sulfoxylate, demonstrating a narrow distribution of particle size; however it was somewhat broader using hydrazine hydrate as the reducing agent that designates non uniform distribution of particle size. Between these two reducing agents, sodium formaldehyde sulfoxylate offers superior quality of Ag NPs in PVA polymer than hydrazine hydrate. They demonstrated that sodium formaldehyde sulfoxylate was greatly efficient as a reducing agent for Ag NPs creation with narrow size distribution. The Ag NPs development with superior quality was thought to be because of a slow reduction mechanism in comparison to the reduction performed by means of hydrazine hydrate. It was indicated that the mechanism of reduction is relatively rapid using hydrazine hydrate. However; they used chemical substances as a reducing agent which are toxic elements and expensive. In the present work, we used quince leaves as a reducing agent which is costless and environmentally friendly material. 

**Figure 6:**
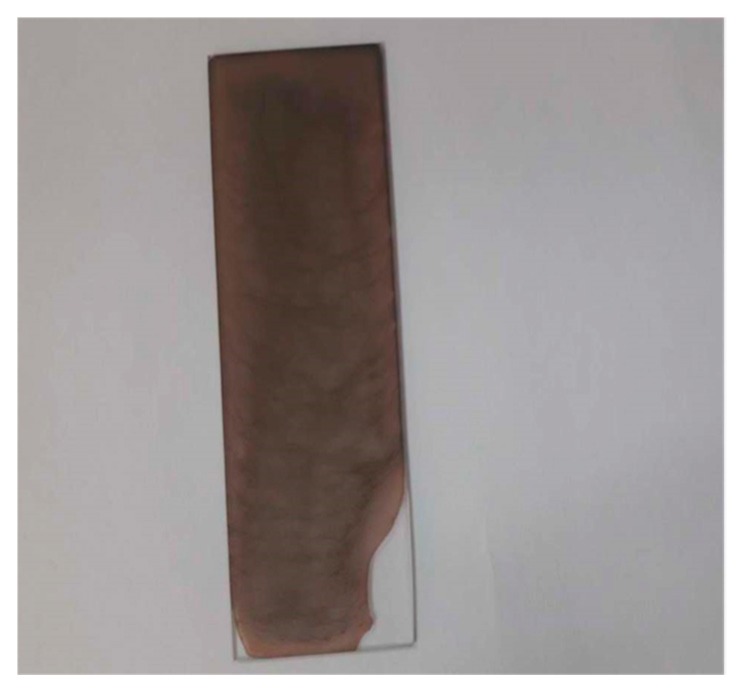
Photograph for deposited silver nanoparticles. The intense brown colour confirms the formation of Ag nanoparticles (NPs).

Figure 6 puts forward the digital photographs that demonstrate that the deposited metallic silver NPs had a deep brown colour. Various applications founded on Ag NPs SPR peak have been put forward in the previous years, specifically for biosensing, surface-enhanced Raman scattering, as well as plasmon circuitry [70]. An interesting point to observe is that a peak at 280 nm was seen in addition to the LSPR peak because of Ag NPs. It was suggested in a previous research that the peaks that are seen at 280 nm are due to the n-π* transitions [71]. The reason for this may be the –OH and –NH_2_ groups′ large density on the Ag NPs surface, as has been determined by FTIR analysis (see Figure 2). 

### 3.4. Morphological Study

**Figure 7:**
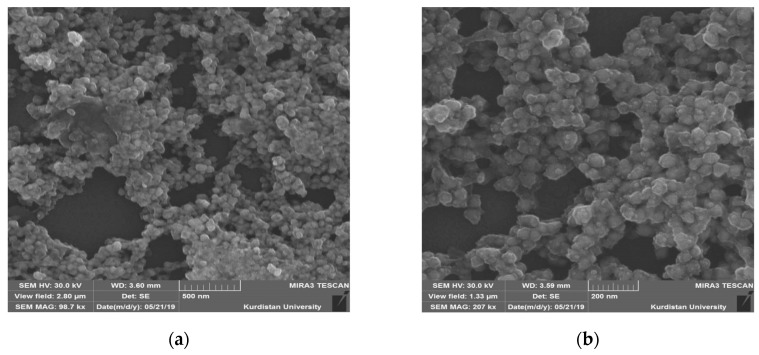

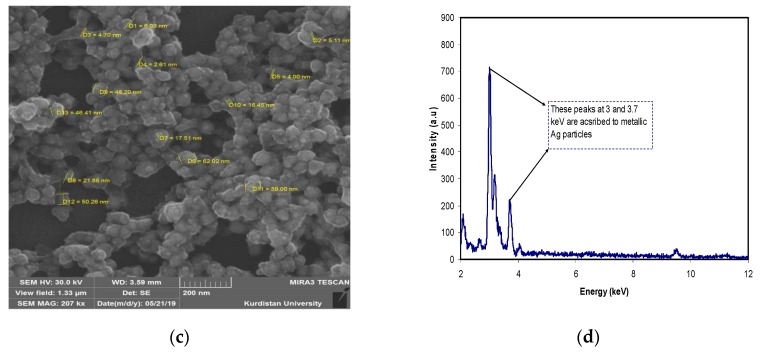
Emission scanning electron microscopy images (FESEM) for (**a**) magnification (MAG): 98.7 kx, (**b**) MAG: 207 kx, (**c**) size of Ag NPs at MAG: 207 kx, and (**d**) energy dispersive analysis of X-rays.

Field emission scanning electron microscopy (FESEM) is a robust technique for identifying the morphological appearance of Ag NPs [72,73,74,75]. Figure 7 demonstrates the surface morphologies and energy dispersive analysis of X-rays (EDAX) outcomes of interconnected Ag NPs. The solution colour change from transparent to brown is indication for the Ag NPs synthesis [76]. Furthermore, the diameter of the majority of Ag NPs was about 50 nm that was greater than those of the Ag NPs colloid investigated by dynamic light scattering DLS and TEM. This phenomenon is due to the Ag NPs large surface energy on the glass slide, which causes the agglomeration of Ag NPs [54].The FESEM images show the dispersion of agglomerated clusters that were distributed with a quite large random of empty space over the surface. This may impact the optical performance of the synthesized structure for real applications, but it is worth noting that the Ag NPs cover the entire region of the visible light as observed in the UV–Vis investigation. It is obvious that the layer of Ag NPs is primarily consisted of uniformly distributed interconnected spherical Ag NPs with high densities at high magnification (see Figure 7b) that cover the surface and no cracks can be observed. Alwan et al. [77] detected numerous cracks in their study on deposited Ag NPs. Furthermore, Schneidewind et al. [78] also investigated Ag NPs on glass substrates. In summary, the SEM images robustly verify the best coating of Ag NPs on the surface of the glass slide. The uniform dispersity of Ag NPs with high densities on glass slide was confirmed from the SEM images. The energy dispersive analysis of X-rays (EDAX) employed for the sample is demonstrated in Figure 7d that indicates the presence of considerable of Ag NPs. The noticeable intense peaks of Ag NPs emerged in the spectrum of EDAX at around 3 and 3.7 keV verified the Ag NPs creation [60,72,79].

## 4. Conclusions

In conclusion metallic Ag NPs were created via the green method. Silver ions reduction to Ag NPs was attained with eco-friendly and costless quince leaves and it can be considered as a clean, low-cost, and safe method which did not use any toxic elements and therefore, has no side effects. The current work verifies the capability of extract solution of quince leaves for the green creation of Ag NPs. These nanoparticles may possibly be used in photonics and optoelectronic devices like ultrafast optical switches. The broad absorption of Ag NPs that covered almost the entire region of visible light in the present work is crucial from the viewpoint of plasmonic solar cells. FTIR examination was performed to recognize potential biomolecules that were accountable for silver ions reduction. Most of the peaks emerged in FTIR pattern of quince leave extract emerged again in the FTIR pattern of Ag NPs but with low intensity. Based on FTIR outcomes schematically silver ion reduction was discussed in detail. In addition, XRD investigation proved the crystalline type of the Ag NPs through the emergence of definite peaks related to Ag NPs. The SPR peak emerged at 480 nm verified the Ag NPs synthesis. The morphology and Ag NPs grain size were attained from FESEM images. Moreover, FESEM images displayed that Ag NPs have interconnected spherical morphology with some aggregations. Intense peaks because of the Ag element emerged at about 3 and 3.7 keV in the EDAX spectrum.
